# Decreasing Psychiatric Emergency Visits, but Stable Addiction Emergency Visits, During COVID-19—A Time Series Analysis 10 Months Into the Pandemic

**DOI:** 10.3389/fpsyt.2021.664204

**Published:** 2021-07-13

**Authors:** Anders Håkansson, Cécile Grudet

**Affiliations:** ^1^Department of Clinical Sciences Lund, Psychiatry, Faculty of Medicine, Lund University, Lund, Sweden; ^2^Region Skåne, Department of Psychiatry Malmö-Trelleborg, Malmö Addiction Center, Malmö, Sweden

**Keywords:** COVID-19, emergency psychiatry, mental health, substance use disorder, treatment seeking

## Abstract

**Background:** The COVID-19 pandemic has been suspected to increase mental health problems, but also to possibly lead to a decreased treatment seeking, for example due to fear of attending hospital. Early findings demonstrate decreased treatment seeking for mental health, which may differ across diagnostic groups. This study aimed to examine treatment uptake at a general psychiatry emergency unit and at an addiction psychiatry emergency unit in Malmö, Sweden, separately. In addition, the study aimed to investigate treatment uptake for different diagnostic groups—during and prior to the COVID-19 pandemic.

**Methods:** Monthly data for number of unique patients and number of contacts were extracted for the three-year period of January 2018 through December 2020. Data from each facility were analyzed separately for women, men and patients with psychotic, affective, anxiety and substance use-related disorders. Interrupted time series were used to demonstrate possible effects of COVID-19.

**Results:** COVID-19 was associated with a marked decrease in treatment contacts, both for women and men, in the general psychiatry emergency unit—driven by a significant decrease in anxiety-related disorders (*p* < 0.001) and affective disorders (*p* < 0.01)—but not in psychotic or substance use disorders (SUDs). Also, in the addiction psychiatry emergency unit, no significant impact of COVID-19 was seen.

**Conclusions:** COVID-19 may decrease treatment uptake for acute affective and anxiety-related disorders. Given the hypothesized increase in the population regarding these conditions, societal efforts are needed to facilitate adequate treatment for these patients during the COVID-19 pandemic. Society should also remain vigilant with respect to SUDs during the pandemic.

## Background

The COVID-19 pandemic, in addition to its devastating consequences on mortality and physical health, has been suspected to have significant effects on mental health worldwide (1). Based on experience from both the COVID-19 pandemic, and from previous pandemics, affective and anxiety-related mental health symptoms are believed to increase as consequence of worsening socio-economic conditions and substantial societal restrictions (2).

However, somewhat paradoxically, despite fears of increased mental health problems during the pandemic, there are also reasons to believe that treatment seeking for psychiatric problems may decrease due to COVID-19 and COVID-19-related restrictions in society. Concerns have been raised about the influence of the pandemic on psychiatric emergency settings (3). Several reports have indicated a lowered number of treatment-seeking patients, for example, in psychiatric units in France or Spain (4, 5), in a German psychiatric hospital (6), a decreasing number of psychiatric presentations at a general emergency unit in New Zealand (7). Also, reductions in treatment seeking have been associated with periods of lock-down or confinement (4, 5, 7).

Actions taken by the government to avoid further viral transmission differ between countries. Many countries have used measurements such as lock-down or stay-at-home orders. However, in the present study setting, Sweden, formal lock-down procedures and complete stay-at-home orders have not been applied as COVID-19-preventive strategies. Instead, strategies have involved national authority recommendations to work at home to the largest extent possible, to avoid public gatherings, to avoid meeting new people and to restrict one's social contacts to the closest family. While such recommendations may decrease mental health patients' willingness to attend hospital and seek treatment, the societal restrictions imposed by Swedish authorities have been described as markedly less strict than in many other countries (8–11).

Addictive disorders are among the conditions believed to increase during COVID-19 (12). Early reports during the pandemic demonstrated that effects on acute psychiatric emergency contacts may differ depending on whether contacts are related to addictive disorders or not, although results have been conflicting so far. One study demonstrated that cannabis use was one of the factors associated with treatment seeking still taking place, despite a decrease in the overall treatment-seeking at a psychiatric emergency unit. However, other specific substances, as well as the overall diagnosis of a substance use disorder (SUD), did not differ during COVID-19 (13). In another study from Western Australia, both alcohol intoxications and “drug abuse” decreased as underlying causes of seeking emergency psychiatric care, whereas the number of drug overdoses remained stable (14). Among self-harm-related emergencies in an Irish study, SUD as diagnosis constituted a significantly higher proportion of patients during 2020 than in the preceding years (15). Likewise, although decreasing in absolute numbers, SUDs represented a larger proportion of psychiatric admissions at a psychiatric emergency unit in Spain during COVID-19 lockdown (5). Thus, it is possible that SUD-related treatment needs and treatment-seeking behavior remain more stable during the pandemic, although data are hitherto inconclusive and limited. In addition, reports on changes in treatment seeking included, for natural reasons, a relatively brief period of the pandemic. Studies available to date covers time-periods between ~2 to 4 months before and after restrictions such as confinement or lock-down (4–6, 14, 15). The few studies available up to now, addressing a limited time-period of the COVID-19 pandemic, call for longer follow-up investigations of possible impact of COVID-19 on psychiatric treatment uptake.

For these reasons, the present study aimed to study effects of COVID-19 on treatment seeking behaviors at a psychiatric emergency unit setting during a substantially longer time frame than previous studies on the subject, namely involving the whole period of COVID-19 impact hitherto in Sweden. Patients' tendency to seek treatment for mental health conditions during COVID-19 may be better reflected in the data of some units than others; for example, long-term, planned treatment interventions at a facility may be less likely to demonstrate whether there are changes in patients' willingness to seek treatment or to refrain from or postpone treatment seeking. In contrast, units where each contact is unplanned, based on the needs perceived by the patient or perceived by families or others, may better reflect short-term changes in the numbers of people choosing to attend hospital for a mental health conditions or who may theoretically postpone their help seeking due to effects of a pandemic on society. For these reasons, psychiatric emergency units were assessed in the present study, that is, a setting where contacts are initiated by patients or by caregivers around them, on a likely short-term basis and typically not occurring as part of a regular long-term planning. The effects of COVID-19 were studied in a separate addiction psychiatry emergency unit and in the remaining general psychiatric emergency unit. Thus, this study had the possibility to analyze the effects of COVID-19 in these settings separately.

More specifically, the study aimed to examine whether the number of unique patients, were affected in these facilities as a result of COVID-19. In addition, for the general psychiatric emergency unit, the numbers of help-seeking patients with SUD were examined. The study aimed to examine these parameters on a month-to-month basis during the full year of 2020, in comparison with the two preceding years. The full year of 2020 represents nearly 10 months of pronounced COVID-19 transmission in Sweden.

## Methods

### Study Procedures

The present study was a retrospective analysis of the number of patients seen in emergency psychiatric facilities in the same catchment area; one general psychiatric emergency unit and one addiction psychiatry emergency unit. The assignment of the addiction psychiatry emergency unit is to receive patients seeking acute in-patient or out-patient interventions, typically involving withdrawal symptoms related to SUDs, attempts to quit substance use, non-life-threatening intoxications of mainly alcohol and psychiatric symptoms in individuals with SUDs. Patients may seek voluntarily, or may be transported by ambulance, police, social workers or staff of other treatment institutions. However, the large majority of contacts are voluntary, whereas a smaller proportion of contacts are initiated while being assessed for compulsory treatment interventions by the social services. This unit operates seven days a week, from 8 am (9 am on weekends) until 11 pm. The general psychiatric emergency unit, operating around the clock, is responsible for all other acute psychiatric treatment seeking, typically involving affective or anxiety symptoms, psychotic symptoms, severe behavioral disruptions or suicidal behavior. For substance-related issues, patients are typically referred to the general psychiatry emergency unit in case of severe suicidal or violent behavior requiring psychiatric coercion and, to a smaller extent, patients seeking help outside the opening hours of the corresponding addiction emergency unit. Patients can, in some cases, be seen in both units when the problem picture is judged to require assessment from both services or when the clinical picture changes during early assessment.

Data of treatment seeking were extracted for each month during the time-period of January, 2018 until December, 2020. Data described the total number of unique patients seen in the facility during each month, including the total number of male and female unique patients, respectively, as well as the total number of contacts per month. Both the numbers of unique patients and the number of separate contacts were included, as some patients may have been seen more than once during 1 month. Thereby, the study limited the risk of separate individuals influencing the statistics with a very high number of visits. In addition, for the general psychiatric emergency unit, the number of unique patients receiving a SUD diagnosis (ICD-10 section F1), psychotic disorders (section F2), affective disorders (section F3) or an anxiety-related disorders (section F4, including disorders such as anxiety, panic disorder, obsessive-compulsive disorders, stress reactions, and phobias), were analyzed. In addition, as descriptive background information for both units, the number of distance contacts were registered, that is, the number of formal assessments made on telephone or video during this time-period.

### Setting

The studied emergency facilities are physically located in the same building in the hospital area of Malmö, which is situated in the urban center of the Skåne region, in the very south part of Sweden. Although a patient can, theoretically, seek psychiatric emergency treatment at any location in Sweden, the natural catchment area of the present two facilities include a population of around 460,000 inhabitants. The two emergency units are responsible for the uptake of unplanned, emergency needs for assessment and treatment for psychiatric conditions in general, and for addiction psychiatry, respectively. The general psychiatry emergency unit is open around the clock, whereas the addiction emergency unit is open from 8 am to 11 pm, and for which the (low) number of individuals seeking during the night are instead referred to the general psychiatry emergency unit (the latter is one part of the explanation that patients in the general psychiatry emergency unit may be diagnosed with addictive disorders, although a considerably more common reason for this is comorbidity of addictions and other psychiatric conditions, in patients whose reason for contact is more related to the presentation of pronounced psychiatric symptoms). The general psychiatry emergency unit typically receives patients with acute suicidal ideation or suicidal behavior, acute worsening of affective and anxiety disorders conditions, psychotic episodes, self-harm or states of confusion. The addiction psychiatry emergency unit receives patients for acute needs for withdrawal treatment, assessment for potential in-patient detoxification, or other types of acute worsening of pre-existing addictive disorders. Both units operate on the same hospital area as the main emergency unit for medical and surgical conditions for adults, although in separate buildings. Both the psychiatric units assessed in the present work are aimed to adults, that is, for individuals above 18 years of age.

**Figure 1:**
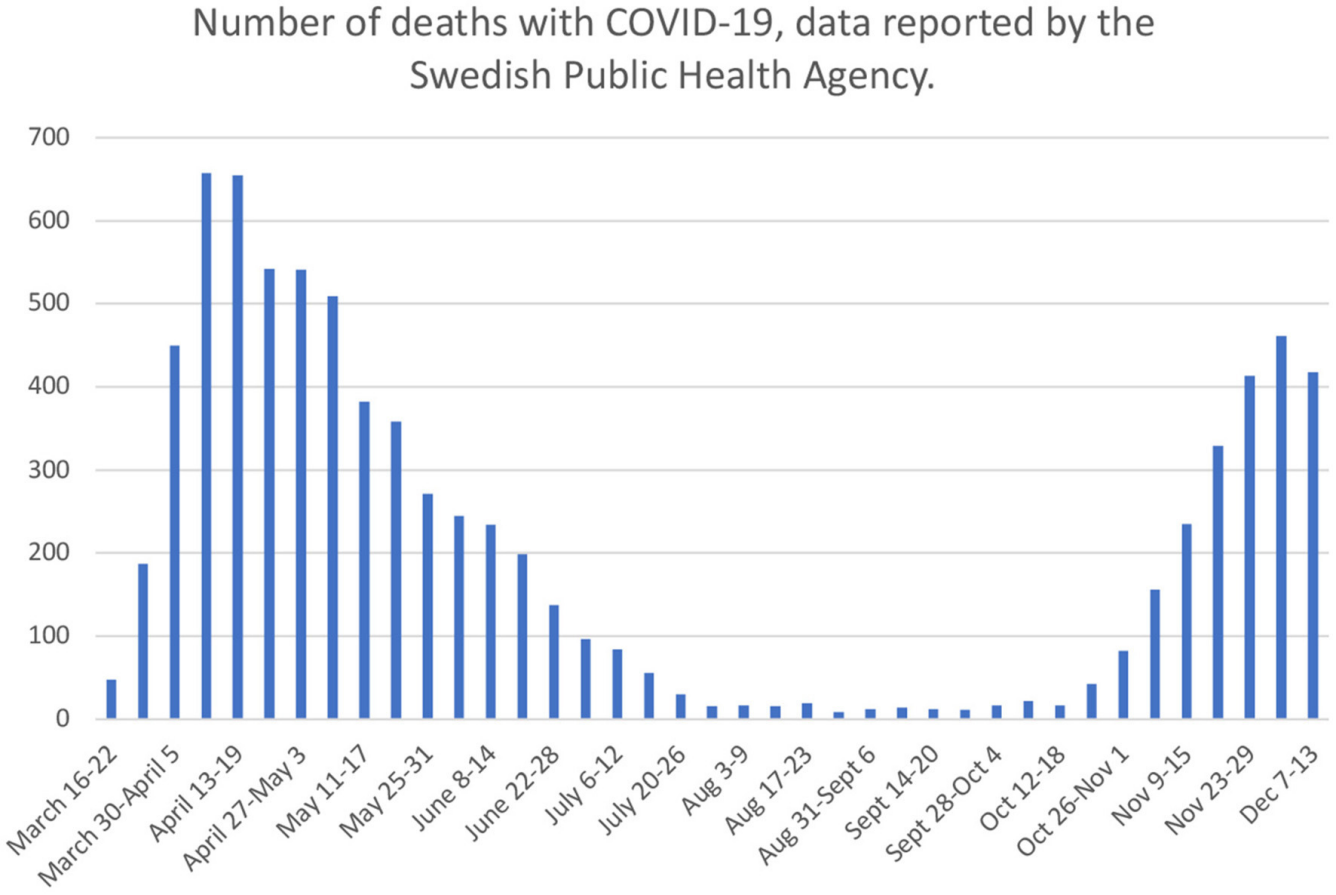
COVID-19 development during 2020. Number of deaths with confirmed COVID-19 infection. Data collected from official statistics of Swedish Public Health Agency (https://www.folkhalsomyndigheten.se/contentassets/4b4dd8c7e15d48d2be744248794d1438/jamforelse-av-olika-matt-pa-covid-19-dodsfall.pdf). Data accessed February 4, 2021.

The course of COVID-19 influence in the Swedish society is demonstrated in Table 1. Also, the official numbers of deaths in Sweden (per week) with confirmed COVID-19 infection are seen in Figure 1 (data derived from the official web page of the Swedish Public Health Agency). In brief, an increasing debate surrounding COVID-19 was seen in early March, 2020 and a large number of restrictions and recommendations were implemented in mid-March. After substantial virus transmission throughout May, the burden on the hospital system was substantially lower during the summer months. Thereafter, a marked surge in COVID-19 cases was seen from mid-October, which continued with a high level of virus transmission and health care impact at least through November and December 2020 (16). During 2020, formal lock-down measures or stay-at-home orders were never applied in Sweden, where COVID-19 policies instead focused on recommendations, such as avoiding public gatherings, avoiding meeting new acquaintances, maintaining thorough hand washing, to stay at home to the largest extent possible and to take a COVID-19 test in case of suspected symptoms. The Swedish authority recommendations have been described as less strict than in a number of other locations (8–10) and Sweden is one of few comparable countries where a formal lockdown procedure was never applied (17). Many government recommendations were issued and applied during the spring months of 2020, based on the first “wave” of intense viral transmission and fatal cases. Recommendations from October, 2020, related to a second “wave,” were more extensive and were in progress throughout the study period.

**Table 1:** Course of the COVID-19 pandemic in Sweden.

	**Date and event**
January 31	First confirmed COVID-19 case in Sweden
February 26	Second confirmed COVID-19 case in Sweden
March 10	Government decision to prohibit public gatherings or more than 500 people
March 11	First confirmed fatality with COVID-19
March 14	Government advice to avoid abroad traveling
March 16	Authority recommendation to work at home, and for individuals aged above 70 years to stay at home
March 17	Recommendation to high schools and universities in Sweden to conduct their studies online
March 27	Government prohibition of public gatherings of more than 50 people
April 6–12	Highest “first wave” number of weekly deaths with COVID-19 (657 deaths per week)
August 24–30	Lowest “between-wave” number of weekly deaths with COVID-19 (9 deaths per week)
October 27	Enhanced regional COVID-19 recommendations in Skåne region (second region in the country enhancing new restrictions)
November 2–8	Large increase in weekly number of deaths with COVID-19 (156 deaths per week)
November 16	Government prohibitions against formal public gatherings of more than eight people, and advice for an eight-people limit to be normative in private life
November 30-December 6	New top “second wave” level of weekly deaths with COVID-19 (461 deaths per week)
December 3	Authority recommendation for high schools to re-introduce distance teaching
December 8	Authority recommendation to celebrate Christmas and other holidays in a very limited group of people
December 14	Text message sent to all mobile telephone, emphasizing the need to follow authority recommendations in Sweden
December 18	Strong authority recommendation to merchants to avoid Christmas sale and similar campaigns
December 22	Closing of border from United Kingdom and Denmark due to new virus mutation
December 27	First COVID-19 vaccine given in Sweden

*Key dates indicating major events and societal adaptations to the pandemic*.

### Ethical Considerations

An application was submitted to the Swedish Ethical Review Authority, regarding the addiction psychiatry emergency unit (along with other units of the Malmö Addiction Center, Malmö, Sweden). The announcement of the authority was that the present kind of research does not require ethical permission since it only examines retrospective clinical register data on a group level and uses no information which can be linked to identified individuals (file number 2020-03232). Based on this decision, the study was carried out and data involving the corresponding unit of the general psychiatry department were added.

### Statistical Methods

An interrupted time series analysis was carried out for each outcome measure (number of patients, number of men, number of women, number of contacts, and number of SUD patients in the general psychiatry emergency unit), analyzing the potential effects of COVID-19 over time (per month during the 3-year study period) when controlling for time. The analyses included a dichotomous factor describing the potential COVID-19 effect, which was considered to start in March, 2020 (thus, with the COVID-19 item represented by “1” for each of these months, and “0” for each of the preceding months). A time variable describing months (January, 2018–December, 2020, 36 months) was entered in the analyses. Analyses were carried out as an interrupted time-series analysis using the ARIMA models in the forecasting tools of the software SPSS version 25.0.

In addition, in order to decrease the possible influence of seasonality, a Student's *t*-test was run for the outcome measures, studying only the COVID-19-affected months (March through December, 2020) in comparison to the same months during previous years (March through December) of 2018 and 2019 combined. The *t*-test analyses included only the numbers of visits (contacts), as the numbers of unique patients may include individuals with a registered visit during more than 1 month. All analyses were carried out in SPSS version 25.0. Associations with a *p*-value below 0.05 were considered to be significant.

## Results

**Figure 2:**
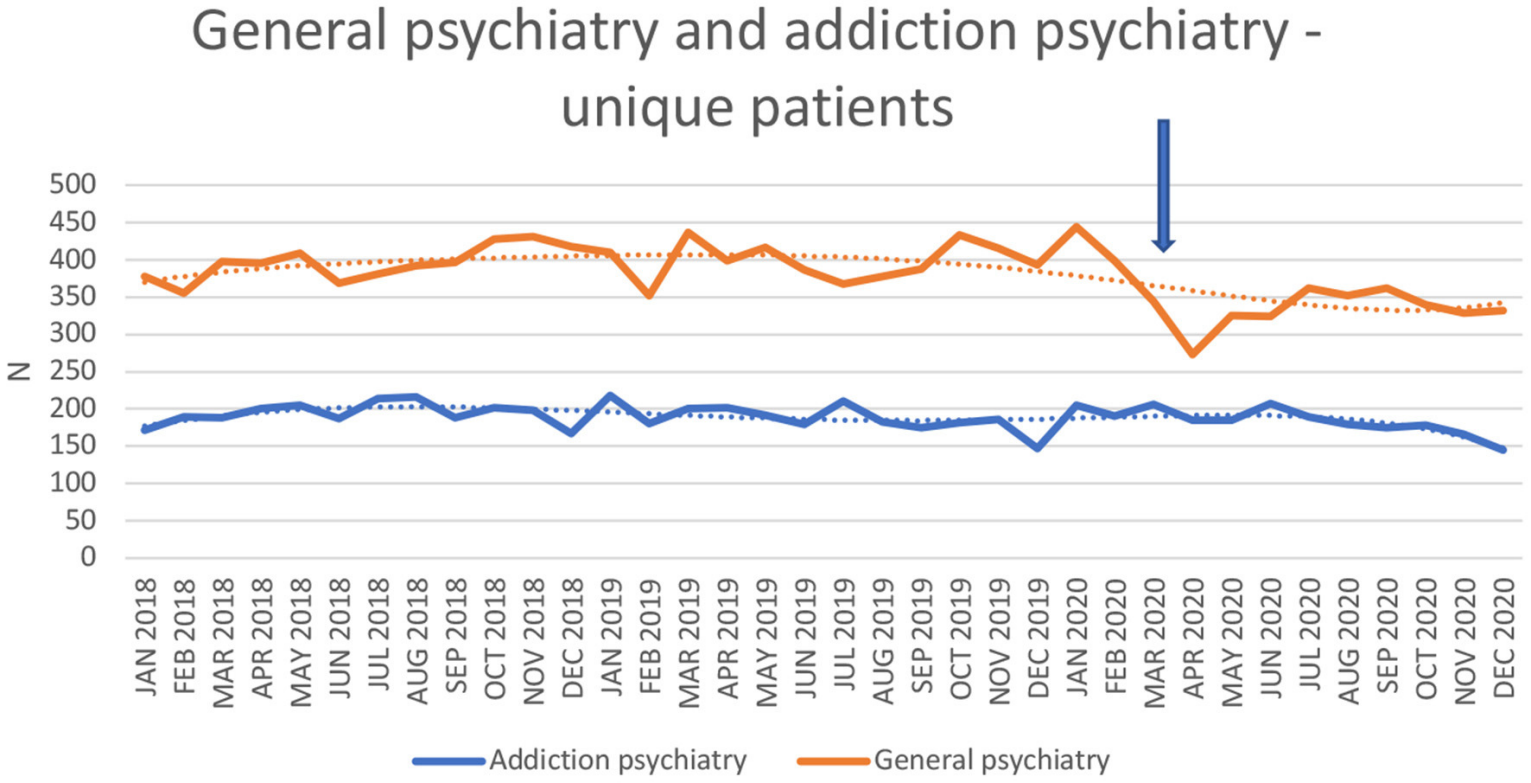
Number of unique patients in general emergency psychiatry and addiction emergency psychiatry.

**Figure 3:**
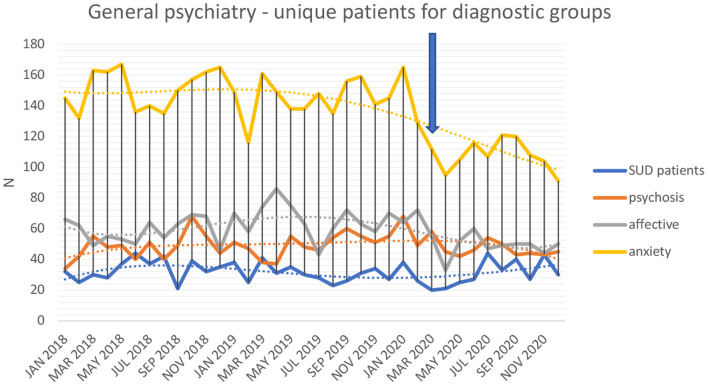
Number of unique patients in general emergency psychiatry with diagnoses of substance use disorders, psychotic disorders, affective disorders, and anxiety-related disorders.

Data on treatment-seeking unique patients (total, and men and women separately), as well as the total number of contacts, are seen in Table 2 (for general emergency psychiatry) and in Table 3 (for addiction emergency psychiatry). Data on treatment-seeking unique patients in each unit are displayed visually in Figure 2, and data on diagnostic groups at the general emergency psychiatry unit in Figure 3.

**Table 2:** Psychiatry emergency contacts in Malmö psychiatric emergency unit, 2018–2020.

	**Unique patients**	**Total contacts**	**Unique patients, men**	**Unique patients, women**	**Registered distance contacts**
Jan, 2018	378	456	175	203	0
Feb, 2018	355	419	168	187	1
March, 2018	397	493	206	191	0
April, 2018	395	472	178	217	0
May, 2018	409	502	182	227	0
June, 2018	369	458	187	182	0
July, 2018	381	460	176	205	0
Aug, 2018	392	482	186	206	0
Sept, 2018	396	493	183	213	3
Oct, 2018	428	513	199	229	4
Nov, 2018	431	566	206	225	4
Dec, 2018	418	515	194	224	3
Jan, 2019	410	540	192	218	0
Feb, 2019	352	433	188	164	0
March, 2019	437	531	216	221	0
April, 2019	399	461	184	215	1
May, 2019	416	508	194	222	2
June, 2019	386	475	203	183	0
July, 2019	368	450	191	177	0
Aug, 2019	378	447	182	196	0
Sept, 2019	388	482	192	196	0
Oct, 2019	433	511	209	224	0
Nov, 2019	415	496	207	208	0
Dec, 2019	393	482	196	197	0
Jan, 2020	444	549	223	221	1
Feb, 2020	399	492	202	197	2
**March, 2020**	**344**	**446**	**181**	**163**	**1**
**April, 2020**	**273**	**335**	**124**	**149**	**0**
**May, 2020**	**325**	**399**	**163**	**162**	**0**
**June, 2020**	**324**	**392**	**163**	**161**	**0**
**July, 2020**	**362**	**425**	**175**	**187**	**0**
**Aug, 2020**	**352**	**425**	**167**	**185**	**0**
**Sept, 2020**	**362**	**456**	**174**	**188**	**1**
**Oct, 2020**	**340**	**419**	**161**	**179**	**0**
**Nov, 2020**	**329**	**401**	**156**	**173**	**0**
**Dec, 2020**	**334**	**388**	**157**	**177**	**17**

*COVID-19-affected months indicated in bold text*.

**Table 3:** Addiction psychiatry emergency contacts in Malmö addiction psychiatric emergency unit, 2018–2020.

	**Unique patients**	**Total number of contacts**	**Unique patients, men**	**Unique patients, women**	**Registered distance contacts**
Jan, 2018	172	263	135	37	0
Feb, 2018	189	297	134	55	0
March, 2018	188	298	138	50	0
April, 2018	201	317	149	52	0
May, 2018	205	320	148	57	0
June, 2018	187	295	140	47	0
July, 2018	214	346	156	58	0
Aug, 2018	216	379	163	53	0
Sept, 2018	188	336	139	49	0
Oct, 2018	202	335	155	47	0
Nov, 2018	198	328	151	47	0
Dec, 2018	167	280	128	39	0
Jan, 2019	218	472	166	52	3
Feb, 2019	180	343	133	47	7
March, 2019	200	473	147	53	15
April, 2019	202	413	156	46	21
May, 2019	192	388	137	55	14
June, 2019	179	333	132	47	17
July, 2019	210	439	157	53	21
Aug, 2019	183	335	138	45	15
Sept, 2019	175	308	130	45	19
Oct, 2019	182	334	141	41	14
Nov, 2019	186	343	146	40	16
Dec, 2019	147	288	104	43	14
Jan, 2020	205	477	153	52	12
Feb, 2020	190	425	135	55	27
**March, 2020**	**206**	**468**	**158**	**48**	**28**
**April, 2020**	**185**	**330**	**139**	**46**	**34**
**May, 2020**	**185**	**388**	**133**	**52**	**16**
**June, 2020**	**207**	**375**	**156**	**51**	**22**
**July, 2020**	**189**	**320**	**139**	**50**	**26**
**Aug, 2020**	**179**	**322**	**120**	**59**	**17**
**Sept, 2020**	**175**	**309**	**125**	**50**	**19**
**Oct, 2020**	**178**	**304**	**123**	**55**	**26**
**Nov, 2020**	**166**	**315**	**122**	**44**	**21**
**Dec, 2020**	**145**	**274**	**112**	**33**	**10**

*COVID-19-affected months indicated in bold text*.

### Interrupted Time-Series Analysis of Number of Visits and Unique Patients at Emergency Units

In interrupted time-series analyses, controlling for general time trends throughout the study, the COVID-19 factor had a significantly negative effect on the monthly number of unique patients (*p* < 0.001) and number of treatment contacts (*p* < 0.001) in the general psychiatry emergency unit. This significantly negative effect was seen both for the number of unique male (*p* < 0.001) and female patients (*p* < 0.001). However, the number of unique patients (*p* = 0.57) and the number of contacts (*p* = 0.57) related to a SUD diagnosis were not affected by COVID-19. The number of unique patients with psychosis diagnosis was not affected (*p* = 0.52) by the pandemic, whereas the number of patients with affective disorders decreased (*p* < 0.01), as well as the number of patients with anxiety-related disorders (*p* < 0.001).

In contrast, in the addiction psychiatry emergency unit, COVID-19 did not have any significant effect on the number of unique patients (*p* = 0.30), the number of contacts (*p* = 0.67), or the number of unique male (*p* = 0.10) or female patients (*p* = 0.65).

Both units applied distance contacts to some extent during COVID-19; in addiction emergency psychiatry, this was also used during 2019 and did not increase significantly with COVID-19 (*p* = 0.48). In psychiatric emergency, distance contacts were used to a very limited extent prior to the pandemic and expanded as late as in December, 2020 (with a non-significant effect from COVID-19, *p*=0.51).

### *T*-Test Analyses of Number of Contacts During COVID-19 (March-December, 2020) vs. Preceding Years

During the months of March through December, 2020, in general emergency psychiatry, the number of unique patients (334.5 vs. 401.5), total contacts 408.6 vs. 489.9), unique female (172.4 vs. 207.9) and male patients (162.1 vs. 193.6) were significantly lower (all *p* < 0.001) than in the corresponding months of 2018-2019, whereas the numbers of patients (31.0 vs. 32.6, *p* = 0.62) or contacts (33.9 vs. 35.6, *p* = 0.64) with SUD were not lower. In addiction emergency psychiatry, the year of 2020 did not display any difference with respect to the number of patients (181.5 vs. 191.1, *p* = 0.18), total contacts (340.5 vs. 343.3, *p* = 0.89), unique male (132.7 vs. 142.8, *p* = 0.10) or female patients (48.8 vs. 48.4, *p* = 0.86). Distance contacts were significantly higher (21.9 vs. 8.3, *p* < 0.001) in addiction emergency psychiatry, but not in general emergency psychiatry (1.9 vs. 0.9, *p* = 0.55).

### Sensitivity Analysis of Planned Out-Patient Contacts

At the two major out-patient general psychiatry units, when comparing the ten COVID-affected months to the same months (March through December) of 2018 and 2019 combined, a difference in the mean monthly number of visits was not seen (*p* = 0.11). Also, an interrupted time-series analysis did not reveal any COVID-19-related impact on the monthly number of patients (*p* = 0.17) or visits (*p* = 0.34) at the two major out-patient general psychiatry units combined. The mean monthly number of visits related to affective disorders did not differ between the 10 COVID-19-affected month compared to the same months in 2018 and 2019 (*p* = 0.40), whereas the mean monthly number of visits related to anxiety disorders was lower (727 vs. 849 visits, *p* = 0.04). However, in interrupted time-series analyses, there were no significant COVID-19-related impact on the monthly numbers of patients (*p* = 0.33) or visits (*p* = 0.53) related to affective disorders, but a marginally significant association with a lower number of patients (*p* = 0.05), but not visits (*p* = 0.21), related to anxiety disorders.

## Discussion

The present study provided diverse trends in COVID-19-related effects on treatment seeking behaviors in general emergency psychiatry and addiction emergency psychiatry. Altogether, general emergency psychiatry had a (significantly) decreased treatment uptake, with significant decreases seen for affective and anxiety-related diagnoses, whereas treatment seeking in the addiction emergency psychiatry unit (as well as for addiction-related problems in general emergency psychiatry) did not decrease. While the decreases in treatment seeking were seen for conditions where a corresponding decrease in planned, non-emergency contacts was not evident, these findings highlight the worrying tendency for patients to refrain from actively seek treatment in emergency phases of a number of common mental health conditions.

Importantly, treatment seeking at the general psychiatric emergency unit dropped significantly during the pandemic. While treatment seeking remained stable for psychotic disorders, it demonstrated a substantial decrease for anxiety-related and affective disorders. Depressive and anxiety-related symptom in the population have been suggested as an early consequence of COVID-19 in several studies (18). In a German web survey, describing self-reported COVID-19 effects on people with different types of self-assessed mental health problems, depression and anxiety were among the disorders reported to have worsened during the pandemic (19). The impacts of COVID-19 on mental health in the clinical setting in Sweden are comparable to impacts shown in previous research in a general population survey in Sweden (20). Importantly, the pandemic seems to impose most on the mental health of those already burdened with the impacts of mental health problems. These results provide a basis for providing more support for vulnerable groups and for developing psychological interventions suited to the ongoing pandemic and similar events in the future. In contrast, however, in recent general population data from the Netherlands; while emotional loneliness increased during the first few months of the pandemic, scoring of clinical symptoms of depression or anxiety did not increase (21). Thus, although still early in the course of health consequences of the pandemic, studies are hitherto not conclusive on the actual mental health consequences. Whether or not an actual change in psychological symptoms has occurred in the population, a possible explanation of the substantial decrease in affective and anxiety-related disorders in the present study may be due to recommendations of social distancing and possible fear of visiting hospital facilities, the latter being suggested in several studies as a reason for avoiding help-seeking (22–24). Also, the present findings related to mental health corroborate with research demonstrating that people may delay or avoid seeking treatment even for acute physical disease, which may generate possible severe consequences (25).

Further, it cannot be excluded that COVID-19 disease itself may have influenced treatment seeking. It was demonstrated in a Korean sample that mental health disorders may be associated with higher virus transmission, although the difference was demonstrated only for psychotic disorders in the study. In addition, among infected individuals, having a mental health disorder was associated with higher mortality (26). A Spanish survey study, describing consequences of lock-down, showed more extensive consequences in patients with mental illness than in community controls. This issue goes beyond the aims of the present study, but emphasizes the need to maintain treatment seeking in patients with poor mental health, as otherwise, patients with higher risks of disease and complications may paradoxically be less prone to seek treatment (27). More research is warranted in order to thoroughly examine the relationship between actual changes in mental health in society and subsequent changes in treatment seeking.

One important finding in the present study was the fact that whilst general emergency psychiatric contacts decreased, hypothetically based on current COVID-19-related restrictions in society, this was not the case for addiction emergency psychiatric contacts. Interestingly, this pattern was seen both for the addiction unit itself and for the sub-sample of patients with SUD within the general emergency psychiatry unit, which may have several explanations. For example, the lack of decrease in addiction-related treatment seeking may reflect a necessity for the patient group to seek treatment due to the severity of the conditions, regardless of fear of virus transmission and/or advices to avoid public gathering. As part of this, it is also possible that addictive disorders may involve a varying degree of formal coercion, fear of such coercion or varying degree of emotional pressure from loved ones around the patient. A third explanation may also be that an actual *increase* in treatment-requiring addictive behaviors may be present, although blunted by a coexisting decrease in treatment seeking behavior and thus leaving an unchanged net effect on treatment seeking data.

The present findings of a stable treatment seeking pattern for acute SUD problems and decreased treatment seeking for depression and anxiety, are to some extent in line with the reports on psychiatric emergencies before and during lockdown in Spain. In a Spanish study, the overall treatment seeking decreased substantially, however, the most pronounced decrease was seen for anxiety disorders. As the absolute number of treatment-seeking SUD patients also decreased, their proportion of the whole patient sample increased during lockdown (5). Possibly, this may corroborate the present findings of SUD requiring more stable treatment needs, even during a period when treatment seeking for mental health overall may be hindered by the pandemic. Thus, given the uncertainty and conflicting findings from previous studies, the present study strengthens the hypothesis that addictive behaviors are among the mental health issues remaining more stable in general emergency psychiatry, despite the effects of the COVID-19 pandemic.

The present study intended to study treatment seeking patterns in a setting where visits typically not part of a regular, long-term planning for mental health conditions, but where a visit is prompted by acute or at least short-term requirements of patients or by their caregivers; that is, the treatment seeking at a psychiatric emergency unit is assumed to involve a certain degree of choice of whether to seek emergency treatment, or to postpone or refrain from treatment seeking. Thus, in this individual decision making about whether to seek help at a given moment or not, hypothetically, reasons related to the particular situation of the COVID-19 pandemic would be part of the decision making. Therefore, it was also of value to contrast these findings to those of the sensitivity analysis, which intentionally measured a setting where psychiatric contacts are typically part of a more long-term procedure and may be based on plans established prior to the pandemic. It is interesting to note that in this sensitivity analysis, in an out-patient setting mainly providing planned contacts as part of an ongoing treatment contact, there were very few significant changes in treatment uptake related to the COVID-19 pandemic. While the total number of general psychiatry patients at these facilities did not change, the number of patients with anxiety disorders, but not affective disorders, did not change, and this was not confirmed in the analysis of actual visits related to anxiety disorders, where no difference was seen. Thus, altogether, this demonstrates that the decrease in emergency contacts in general psychiatry, including in the most common general psychiatry diagnoses specifically, was not clearly seen in the planned out-patient treatment setting. It can be argued that emergency contacts are more likely than regular, planned out-patient contacts to reflect voluntary treatment-seeking behaviors and changes in such behaviors, such as changes related to fear of COVID-19 or altered lifestyle habits related to the pandemic.

The present study may have a number of implications. Authors of a study in the U.S., have suggested a number of interventions potentially useful in the adaptation of psychiatric care to the COVID-19 situation (3). As there is little reason to believe that mental health needs have decreased during the pandemic, the lowered treatment uptake in general emergency psychiatry suggests a need to facilitate access to mental health care for patients who do not seek treatment on different grounds. Also, one implication may be to further study addictive behaviors in the society during the pandemic as the figures from the present study may suggest that addictive diseases may at least be preserved, and possibly increased, during this period. The present findings call for close attention to addictive behaviors in society, throughout and after the COVID-19 pandemic, as these issues did not demonstrate the decrease seen for emergency psychiatry in general.

One further implication highlights the need for increased telemedicine interventions in the emergency settings. Despite the possibility of such contacts, these were very low prior to the pandemic. However, it can be argued from the present data, as well as from the clinical experience in the present setting, that the reasons for assessing patients in an emergency unit, either because of an acute psychiatric conditions or acute treatment needs related to addictive disorders, are unlikely to be replaced to a major extent by distance contacts. Thus, in contrast to out-patient non-emergency settings, where telepsychiatric interventions may play a clearer role, the need for assessment or factors such as suicide risk, risk of violence, states of confusion or psychosis, as well as the physical status of patients in need for withdrawal treatment, are factors which make digital replacements difficult. Thus, the actual area of responsibility of the present two emergency units is likely to explain the low use of distance contacts, both before and even during the pandemic.

The present study is a retrospective longitudinal analysis of treatment uptake in two emergency psychiatric units. As the study was carried out in only one urban area, the findings cannot be readily generalized to other settings. In particular, with respect to COVID-19 restrictions, these are known to have differed between countries and over time, which naturally limits the generalizability of the study to other geographical settings. Generalizability may also be limited by the fact that the role of emergency psychiatric facilities may be different in different settings; the extent to which unplanned help seeking of patients happens at emergency units, in contrast to more traditional out-patient facilities during office hours, may differ across settings.

Moreover, given its retrospective and anonymous data collection, the study has a number of limitations. The study cannot describe information on the severity of conditions, nor can it describe the full picture of secondary diagnoses. Also, as the study did not involve detailed individual hospital records, the data cannot describe whether treatment seeking was voluntary or prompted by families or authorities, or whether patients sought help for poor mental health for the first time or in the picture of a pre-existing condition. Strengths of the study include the fact that the study involves all occasions of treatment seeking at the present facilities during 10 months of the pandemic. We were also able to provide a relatively satisfactory time-period for control previous to COVID-19 outbreak in this setting. Thus, although detailed hospital records of patients are not included, the study is able to describe how patterns of active treatment seeking may have differed over time. Future studies need to provide more in-depth descriptions of the reasons for seeking or not seeking emergency care during COVID-19.

## Conclusions

In conclusion, during a nearly 10-months long period of COVID-19 pandemic in Sweden, treatment uptake in a general psychiatry emergency unit was substantially decreased, reflecting a lowered tendency to seek mental health emergency treatment for some conditions. Barriers against treatment-seeking, such as fear of attending hospital, can be suspected. In particular, the number of patients attending with affective or anxiety-related diagnoses decreased. In contrast, however, addiction emergency treatment seeking remained stable. Our suggestions are that stakeholders should address methods for maintaining treatment seeking in people with poor mental health and that further observations should be made regarding addictive behaviors in society during COVID-19, as these behaviors at least do not seem to decrease.

## Data Availability Statement

The raw data supporting the conclusions of this article will be made available by the authors, without undue reservation.

## Ethics Statement

The studies involving human participants were reviewed and approved by Swedish Ethical Review Authority. Written informed consent for participation was not required for this study in accordance with the national legislation and the institutional requirements.

## Author Contributions

AH and CG were responsible of the overall research idea, data collection, the analyses, and scientific interpretation of the data and made substantial revisions of the draft. AH wrote the manuscript draft. All authors approved the final version.

## Conflict of Interest

AH holds a position as a professor at Lund University which is sponsored by the state-owned gambling operator AB Svenska Spel, which has no role in the present research. Also, the research group has funding from the research council of the state-owned alcohol monopoly, Systembolaget, which also had no role in the present research. The remaining author declares that the research was conducted in the absence of any commercial or financial relationships that could be construed as a potential conflict of interest.
